# Terminal chromosome 4q deletion syndrome in an infant with hearing impairment and moderate syndromic features: review of literature

**DOI:** 10.1186/1471-2350-15-72

**Published:** 2014-06-25

**Authors:** Barbara Vona, Indrajit Nanda, Cordula Neuner, Jörg Schröder, Vera M Kalscheuer, Wafaa Shehata-Dieler, Thomas Haaf

**Affiliations:** 1 Institute of Human Genetics, Julius-Maximilians-Universität Würzburg, Biozentrum, Am Hubland, 97074 Würzburg, Germany; 2 Department of Human Molecular Genetics, Max Planck Institute for Molecular Genetics, Berlin, Germany; 3 Comprehensive Hearing Center, Department of ORL, Plastic, Aesthetic and Reconstructive Head and Neck Surgery, Würzburg, Germany

**Keywords:** Genotype-phenotype association, Copy number variation, Parent-of-origin, SNP array, Terminal 4q deletion syndrome

## Abstract

**Background:**

Terminal deletions of chromosome 4q are associated with a broad spectrum of phenotypes including cardiac, craniofacial, digital, and cognitive impairment. The rarity of this syndrome renders genotype-phenotype correlation difficult, which is further complicated by the widely different phenotypes observed in patients sharing similar deletion intervals.

**Case presentation:**

Herein, we describe a boy with congenital hearing impairment and a variety of moderate syndromic features that prompted SNP array analysis disclosing a heterozygous 6.9 Mb deletion in the 4q35.1q35.2 region, which emerged *de novo* in the maternal germ line.

**Conclusion:**

In addition to the index patient, we review 35 cases from the literature and DECIPHER database to attempt genotype-phenotype correlations for a syndrome with great phenotypic variability. We delineate intervals with recurrent phenotypic overlap, particularly for cleft palate, congenital heart defect, intellectual disability, and autism spectrum disorder. Broad phenotypic presentation of the terminal 4q deletion syndrome is consistent with incomplete penetrance of the individual symptoms.

## Background

Terminal deletions of chromosome 4q are a rare event with an approximate incidence of 1 in 100,000 [1,2]. While the majority are *de novo* cases, an estimated 10-20% are the unbalanced product of a parental reciprocal translocation. Furthermore, some pediatric cases with classical phenotypes have inherited their 4q deletion from a parent described as either normal or only mildly affected [3-6]. Although there is a high degree of phenotypic variation in those presenting overlapping deletion intervals, there is a general consensus that chromosome 4q deletion syndrome is characterized by intellectual disability (ID), craniofacial dysmorphism, rotated or low-set ears, cleft palate (CP), micrognathia, congenital heart defects (CHD), craniofacial, skeletal and digital abnormalities, and occasionally autism spectrum disorder (ASD), behavioural disorders, and developmental delay [7-9]. Chromosome 4q deletions are divided in two different subgroups depending on the region of 4q that is deleted: interstitial, spanning the centromere through 4q28.3 and terminal, from 4q31.1 to 4qter. Although both deletion types each have highly variable phenotypic associations, terminal deletion cases present a broader phenotypic range including CHD, craniofacial and skeletal abnormalities. The 4q33 region has been proposed as critical for ulnar deficiency, cleft lip and palate, and brain development [10].

Herein, we report on an eight year-old boy with moderate dysmorphic features and a *de novo* deletion in the 4q35.1q35.2 region. By analyzing the considerable phenotypic variability of terminal 4q deletion cases from the literature and DECIPHER database, we attempt to delineate critical intervals for common phenotypic features.

## Case presentation

### Clinical report

The proband is the only child of two healthy unrelated parents of German ethnicity, born at a gestational age of 38.3 weeks, after an uncomplicated pregnancy and normal spontaneous delivery. Birth weight was 3,125 g (25^th^ centile), APGAR scores of nine and ten at one and five minutes, respectively, cord blood pH was 7.3, and an unremarkable otoacoustic emissions newborn hearing screening test was recorded. At four months of age, he had bilateral hearing impairment in the 60 dB range and was fitted with hearing aids. We sequenced genes commonly screened for hearing loss, including *GJB2* (MIM: 121011), *GJB3* (MIM: 603324), and *GJB6* (MIM: 604418). Sequencing disclosed a heterozygous mutation in *GJB3* c.94C > T, p.Arg32Trp (rs1805063; minor allele frequency T = 0.015), which is a well-described autosomal recessive deafness gene requiring a second heterozygous mutation either *in trans* or in compound heterozygous configuration to convey hearing loss. A targeted deafness gene next generation sequencing panel was negative for other pathogenic mutations.

In the first year of life, he was diagnosed with aortic isthmus stenosis, corrected via balloon angioplasty, and a patent foramen ovale. He demonstrated shortened PQ intervals on an electrocardiogram indicative of an atrioventricular node irregularity. Regular pediatric cardiology follow-up was recommended. He also presented with chronic Eustachian tube dysfunction that was treated several times with myringotomy tubes, as well as a bifid uvula. In the fifth year of life, a submucous CP was detected. During the same year, he underwent corrective surgery for the CP and velopharyngeal insufficiency. Additionally, he presented with bilateral cryptorchidism that required testicular orchiopexie. An abdominal sonogram could not rule out the possibility of a left duplex kidney; urine analysis was within normal limits. Despite a small thyroid, he had normal thyroid function on lab testing. His blood profile was unremarkable apart from mild concurrent deficiencies of blood coagulation factors IX (56%), XI (48%) and XII (38%). He had an elevated prothrombin time of 46.5 s (normal: 25–39 s) and an elevated lupus anticoagulant confirmatory test of 1.26 (normal: 0.91-1.07). Further coagulation testing was negative for von Willebrand disease.

Psychological developmental evaluation at the age of three to four years showed mild general developmental delay (six months). Subsequent evaluations showed normal development. Neurological evaluation at the age five showed a lack of age-appropriate coordination. He also had delayed speech and language development, likely secondary, at least in part, to his hearing impairment and extensive hospitalization history. Currently, he attends regular school and does not require remedial classroom instruction.

## Methods

### Classical cytogenetic and fluorescence *in situ* hybridization (FISH) analyses

Chromosomes of the proband and his parents were prepared from peripheral blood lymphocyte cultures and analyzed by GTG-banding at the 500 band resolution. FISH was carried out using selected BAC probes from the deleted region. BAC DNA was labelled by nick translocation with fluorescein-12-dUTP (Roche Diagnostics, Mannheim, Germany) or tetramethyl-rhodamine-5-dUTP (Roche), and hybridized overnight to denatured chromosomes. Image acquisition and analysis were performed using FISHView 2.0 software (Applied Spectral Imaging, Edingen-Neckarhausen, Germany).

### Copy number variation and genotype analyses

Genomic DNA (gDNA) of the proband and his parents was prepared from peripheral blood by standard salt extraction method. The Illumina Omni1-Quad v1.0 SNP array (Illumina, San Diego, CA, USA), with >1.1 million SNP markers, was used for whole genome genotyping and copy number variation (CNV) detection. 200 ng gDNA were utilized in an Illumina Infinium HD Ultra Assay according to the manufacturer’s specifications. Data were analyzed using GenomeStudio (v2011.1) software with both cnvPartition 3.2.0 (Illumina) and QuantiSNP 2.2 copy number algorithm [11]. Genotypes of father, mother and proband were obtained from the SNP array for parent-of-origin determination. HaploPainter [12] was used in combination with manual intervention to illustrate the absence of maternal genotypes in the deletion patient. The terminal 4q monosomy was validated by real-time quantitative polymerase chain reaction (qPCR) of *FRG1* exons 1, 8, and *DUX4L6* using the SensiMix SYBR Green kit (Bioline, Luckenwalde, Germany).

### Mapping critical intervals for terminal 4q deletion syndrome phenotypes

This study makes use of data generated by the DECIPHER consortium, which is funded by the Wellcome Trust (http://decipher.sanger.ac.uk). With the combined DECIPHER cases (nos. 278055, 248967, 249192, 249458, 249476, 249536, 249541, 249655, 251175, 253743, 254882, 256186, 257358, 264122, 264942, 267783, 269176, and 276704) and review of the literature [6-10,13-22], phenotypic and deletion overlaps among individuals with monosomies spanning different sizes were delineated. We used the UCSC Genome Browser Custom Track (http://genome-euro.ucsc.edu/cgi-bin/hgCustom) to map these cases and targeted the narrowest critical interval for CP, CHD, ID, and ASD.

## Results

### Classical and molecular cytogenetic analyses

**Figure 1:**
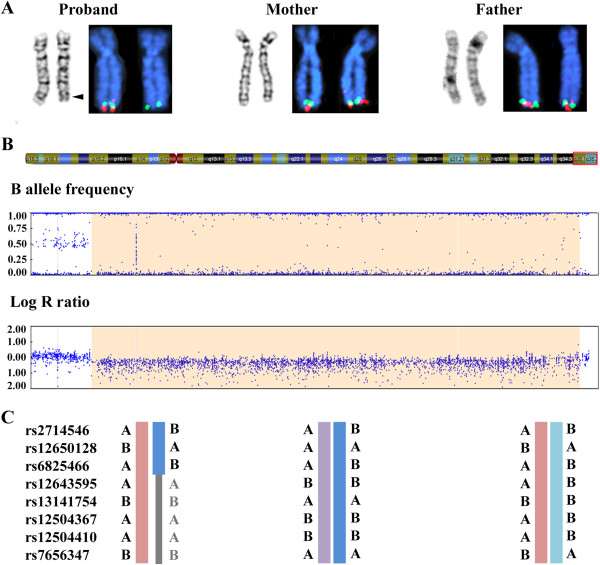
**Molecular karyotyping of the patient and his parents. (A)** GTG-banding and FISH analysis of homologous chromosomes 4 in proband, mother and father. The chromosomes are counterstained with DAPI (blue). The proximal flanking BAC RP11-188P17 is labeled with fluorescein-dUTP (green) and the deleted BAC RP11-775P18 with rhodamine-dUTP (red). An arrowhead indicates the critical band q35.1q35.2 on the patient’s derivative chromosome. **(B)** Illumina SNP array analysis (B allele frequency and log R ratio) of the 4q35.1q35.2 region in the boy with terminal 4q deletion syndrome. **(C)** Selected genotypes in the deletion interval from the Illumina array are depicted for proband (left), mother (middle), and father (right). Mendelian transmission errors (absence of maternal genotypes) in the proband are indicated in gray.

Conventional chromosome banding analysis of the proband revealed a 46,XY karyotype without gross abnormalities. However, the distal G-band negative region in the long arm of chromosome 4 corresponding to q35.1q35.2 appeared to be somewhat smaller in one of the homologs, suggestive of a loss of chromosome material (Figure 1A). Both parents had normal karyotypes without evidence of deletion on chromosome 4q.

To validate the deletion in the proband, SNP array analysis was performed which disclosed a 6.9 Mb heterozygous deletion on chromosome 4q35.1q35.2 (184,046,156-190,901,117 bp from rs17074417 to rs10005101, hg19) (Figure 1B). qPCR analysis of *FRG1* exons 1, 8, and *DUX4L6* confirmed that the distal deletion breakpoint extends beyond 190,939,252 bp (data not shown), encompassing a total of 42 annotated genes (18 OMIM genes). Based on these results, the proband’s karyotype could be assigned as 46,XY,del(4)(q35.1q35.2). SNP array analyses of maternal and paternal DNA did not indicate CNV for chromosome 4q in the parental karyotypes, consistent with a *de novo* deletion in the child. Informative SNPs from the terminal 4q region for which the mother and father have divergent genotypes revealed a loss of maternal genotypes in the child (Figure 1C), compatible with maternal origin of the deleted chromosome.

FISH analysis was performed with BACs from the proximal flanking region 4q35.1 (RP11-188P17) and the deleted region 4q35.1q35.2 (RP11-775P18, RP11-118M15, and RP11-652J12). As expected, the flanking BAC probe hybridized to both chromosomes in the proband and parental metaphase spreads. Probes from the deleted region recognized only one chromosome 4 homolog of the patient, but were present on both chromosome 4q35.1q35.2 copies of father and mother (Figure 1A). Obviously, the *de novo* deletion is not due to a cytogenetically cryptic subtelomeric translocation in a parental karyotype.

### Genotype-phenotype correlation of terminal 4q deletion syndrome

**Figure 2:**
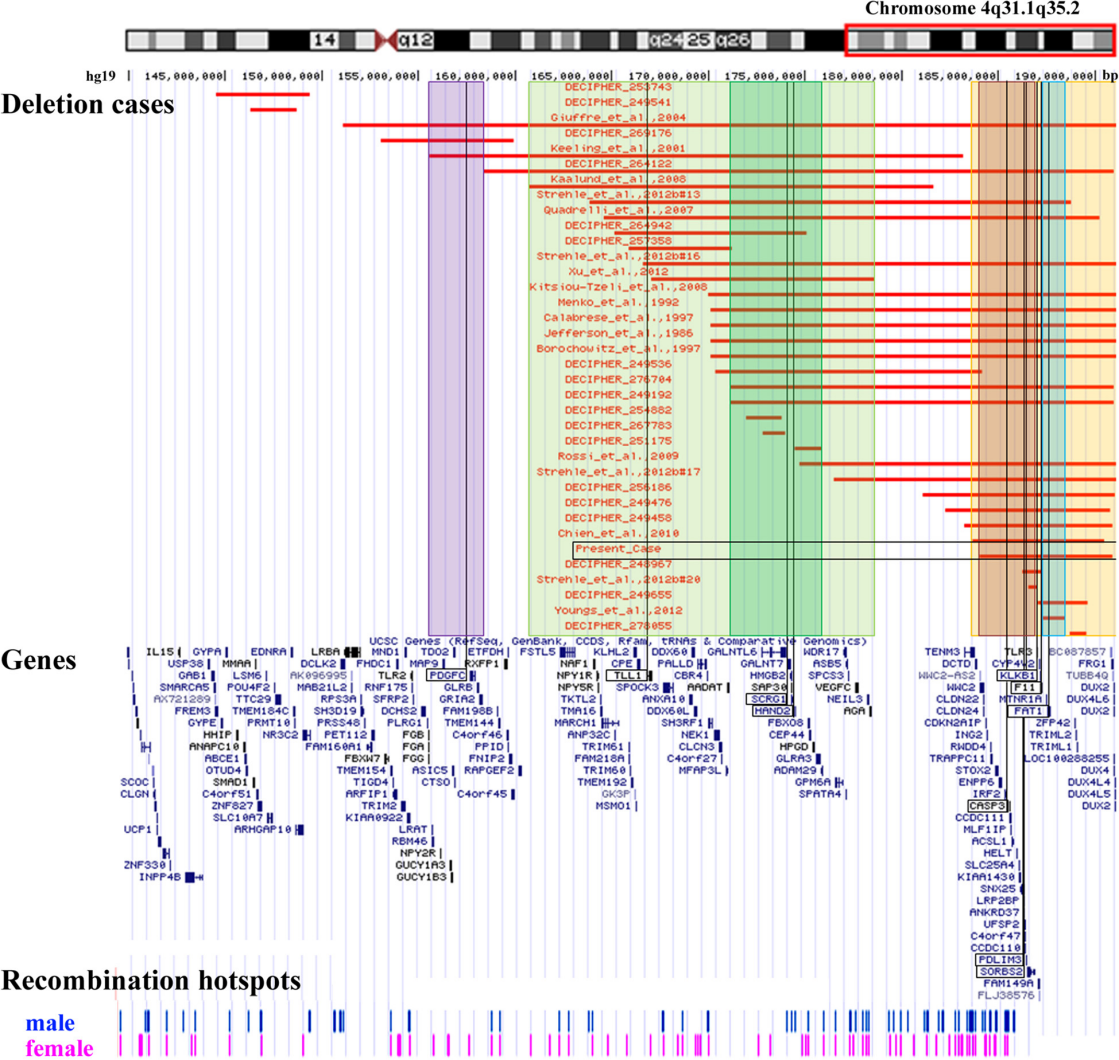
**Genotype-phenotype correlation of terminal 4q deletions.** The upper part of the figure presents the mapped deletion intervals in chromosome 4q31.1qter including the present case, marked with a box, and 35 additional cases from DECIPHER and the literature. The red bars delineate the deletion region for each case. Highlighted intervals indicate critical regions for common phenotypes among the cases. Depicted, from left to right, are the intervals for cleft palate (purple), congenital heart defect region 1 (light green), intellectual disability (dark green), congenital heart defect region 2 (red), and autism spectrum disorder (turquoise). Orange indicates the mapping interval of the DFNA24 locus. The middle section shows the gene content of the 4q31.1qter region. Likely disease-relevant genes overlapping with critical deletion intervals are boxed. The bottom diagram shows the deCODE recombination map, highlighting male and female recombination hotspots in the terminal 4q deletion syndrome region.

We created a map of terminal 4q deletion syndrome cases through reviewing the literature and DECIPHER database. Figure 2 (upper section) presents 36 deletion cases (including our own) meeting our interval criteria and after controlling for normal CNV from the Database of Genomic Variants (http://dgv.tcag.ca/dgv/app/home). Although we had to estimate best fit intervals for cases describing deletions using various low-resolution methods, we were able to roughly map out five critical regions for four common 4q deletion syndrome phenotypes: CP, CHD, ID, and ASD. Figure 2 (middle section) and Additional file 1: Table S1 show the gene content of the deletion region, with an emphasis on genes implicated in the various associated phenotypes. Additional file 2: Table S2 details case summaries including approximate deletion sizes, inheritance and phenotype information.

A locus (chr. 4: 155,600,001-158,373,133 bp) for CP (Figure 2, purple) was mapped with two out of three cases spanning this region having CP [7,10]. The gene *PDGFC* is important for development of the palate with implication in non-syndromic orofacial clefting [23]. Furthermore, *Pdgfc*^-/-^ knockout mice display clefting [24]. Thirteen cases under evaluation indicate CP, suggesting an additional critical interval involved in palate formation.

Congenital heart defects were mapped to two separate regions. The first region (Figure 2, light green) spans a large interval (chr. 4: 160,717,000-178,579,037 bp) unable to be further subdivided based on the cases presented. There are 17 cases with various cardiac phenotypes, 13 of which overlap with the proposed interval, with three individuals unique to this first CHD locus [10,18,21]. This interval contains two genes of interest (Additional file 1: Table S1). *TLL1* is important for mammalian heart septation [25]. Mice with abnormalities in this gene die from blood circulation failure [26]. From mouse and zebrafish experiments, *HAND2* is also involved in cardiac morphogenesis, angiogenesis, and formation of the right ventricle and aortic arch arteries and, interestingly, plays a role in limb formation [27,28]. Although many individuals presented digital and forearm deficiencies, we were not able to clearly map these phenotypes to this region as well.

The second CHD locus (chr. 4: 184,046,156-186,997,806 bp) maps in a region containing 12 out of 17 overlapping cases with cardiac phenotypes (Figure 2, red), two of whom uniquely overlap with this region. The critical interval contains two adjacent genes, *PDLIM3* and *SORBS2*, implicated in cardiac development. *PDLIM3* is essential for right ventricular development and thought to enhance mechanical strength stability of cardiac muscle during mouse development [29]. *SORBS2* is highly expressed in the intercalated disk in normal cardiac tissue [30]. Additionally, *SORBS2* could have implication in CP formation, since case #20 [9] with CP has a small deletion (chr. 4: 186,533,075-186,997,806 bp) exclusively affecting *SORBS2* and *TLR3* (Figure 2, upper and middle section). Ten out of 13 total individuals with CP overlapped with this region, but the proximal border was too large to map an informative locus.

A smaller region (chr. 4: 171,144,641-175,897,427 bp) within the first CHD interval may account for ID*,* with eight of 15 individuals having ID (Figure 2, dark green). While no gene is presently linked to ID in this region, the gene *SCRG1* is highly expressed in the brain and has differential regulation in schizophrenia and bipolar disorder [31] (Additional file 1: Table S1). Lack of genomic variation among healthy individuals in the Database of Genomic Variants and strong evolutionary conservation (data not shown) further emphasize the importance of normal copy number of this ID region.

A number of reports implicate chromosome 4q35.2 in ASD [20,22]. While only four cases reviewed here have ASD (Additional file 2: Table S2), all four overlap one narrow interval (chr. 4: 187,234,067-188,424,639 bp) (Figure 2, turquoise) with only three genes (*MTNR1A*, *FAT1* and *F11*), that was first reported in a boy with ASD [22]. *FAT1* has been associated with bipolar affective disorder [32] and ASD [33], and is essential for controlling developmental cell proliferation [34].

Mild factor XI deficiency and elevated prothrombin time in our proband are presumably explained through deletion of *F11*[35,36] and an adjacent coagulation gene, *KLKB1*[37]. Surprisingly, the mild bleeding tendencies that can be associated with *F11* and *KLKB1* deletions have not been discussed in great detail yet, although many children with terminal 4q deletion syndrome require multiple surgeries.

The first clinical symptom of our patient with a 6.9 Mb deletion (chr. 4: 184,046,156-190,901,117 bp) was mild to severe bilateral hearing loss. Two additional cases with larger deletions, DECIPHER case 256186 and the case from Calabrese et al., 1997 [16], also reported hearing impairment. In a Swiss-German kindred with autosomal dominant non-syndromic hearing loss, an autosomal dominant deafness locus, DFNA24 (MIM: 606282) was mapped to an 8.1 Mb region (chr. 4: 183,200,000-191,154,276 bp) (Figure 2, orange) on chromosome 4q35qter [38,39]. However, in this context it is important to emphasize that 11 normal hearing terminal 4q deletion cases overlap completely and nine normal hearing cases overlap partially with the DFNA24 interval. Thus, loss of one locus copy is not sufficient to cause DFNA24. A cumulative effect of rare, pathogenic variants in different deafness genes scattered across the genome (i.e. haploinsufficiency for DFNA24) could contribute to hearing impairment [40]. Mouse knockout experiments suggest that *Casp3*, which is contained in the critical region, is required for proper functioning of the cochlea [41-43]. *Casp3*^*-/-*^ mice indicated sensorineural hearing loss, whereas *Casp3*^*+/-*^ mice displayed intermediate vestibular dysfunction, as well as marginally increased hair cell counts.

## Discussion

Since its first description [44], the genotype-phenotype delineation of chromosome 4q deletion syndrome has been complicated by extensive inconsistencies reported among individuals with similar deletion intervals. With >170 genes residing in the terminal 4q region, delineation of the phenotypes associated with such deletions presents a tremendous task toward understanding the complete spectral presentation of a syndrome with excessive phenotypic variability. The patient we present was analyzed with a high resolution SNP array to delineate the deletion interval and the parental origin of the *de novo* rearrangement. We found it especially challenging to finely map disease-relevant intervals with the various low-resolution techniques that used GTG banding [7,10,13-15], FISH [16], and the different resolution arrays, including BAC aCGH [18], 1 Mb aCGH [19], 44 K aCGH [6,17,20], 105 K aCGH [9,22], and 300 K SNP array [21]. Another limitation includes possible variations in the depth of clinical descriptions listed, especially those from the DECIPHER database, which were not as detailed as the published cases. Collectively, case-supported critical regions for several distinct phenotypes such as CP, CHD, ID, and ASD were defined. In this context, it is important to emphasize that most phenotypic features that are associated with terminal 4q deletion syndrome show incomplete penetrance and/or are rather unspecific, which renders genotype-phenotype correlations difficult.

The overwhelming majority of cases are *de novo* possibly due to errors during meiotic recombination leading to a loss of chromosomal material from one parental allele. Meiotic crossovers preferentially occur at non-random hotspots which have been mapped according to frequency and spatial distribution in both males and females [45]. The deCODE recombination map [46] of the 4q31.1qter region illustrates an enrichment of both male and female hotspots along the major part of this interval (Figure 2, bottom section). However, it is also possible that deletions arise in mitotically dividing spermatogonial and oogonial stem cells, respectively. The resulting germ-cell mosaicism would increase the likelihood of having another child with the same deletion.

In summary, the case presented here is the first to use a SNP array to determine the parent-of-origin of the large deletion. Assuming that the same gene(s) is underlying hearing impairment in terminal 4q deletion and DFNA24 patients, it may help further narrow the DFNA24 locus. Our case, in combination with the cases described in the literature and DECIPHER, accommodate a proposal of critical phenotypic intervals with possible genes of interest. This review is not intended as a holistic description of terminal chromosome 4q deletion syndrome. However, the ongoing reporting of precisely defined deletion intervals with higher resolution technologies will support eventual refinement and possible clarification of the genes and pathways responsible for the broad phenotypic presentation of deletions in this interval of chromosome 4q.

## Consent

The study was approved by the Ethics Committee of the University of Würzburg. Full informed parental consent was obtained prior to initiating our investigation.

## Abbreviations

ASD: Autism spectrum disorder; BAC: Bacterial artificial chromosome; CHD: Congenital heart defect; CNV: Copy number variation; CP: Cleft palate; FISH: Fluorescence *in situ* hybridization; ID: Intellectual disability; PCR: Polymerase chain reaction.

## Competing interest

No potential competing interest as well as commercial interests were disclosed.

## Authors’ contributions

BV, IN and CN carried out the molecular cytogenetic and data analyses. JS and WSD performed a clinical analysis of the patient. VK provided materials. BV and TH researched the literature and wrote the manuscript. All authors have critically reviewed and approved the manuscript.

## Pre-publication history

The pre-publication history for this paper can be accessed here:

http://www.biomedcentral.com/1471-2350/15/72/prepub

## Supplementary Material

Additional file 1: Table S1 Summary of disease-relevant genes in the deletion region with functions, phenotypes and cases with agreeable phenotypes. Click here for file

Additional file 2: Table S2 Summary of our proband and cases from DECIPHER and the literature with deletions exclusively residing in the 4q31.1qter region. Click here for file
