# Supplementary material for: Terminal chromosome 4q deletion syndrome in an infant with hearing impairment and moderate syndromic features: review of literature
Source: BMC Med Genet. 2014 Jun 25;15:72. doi: 10.1186/1471-2350-15-72 (PMC4077152; doi:10.1186/1471-2350-15-72)
Supplement: Additional file 1: Table S1 — Summary of disease-relevant genes in the deletion region with functions, phenotypes and cases with agreeable phenotypes. [file 1471-2350-15-72-S1.doc]

**Table S1 Summary of disease-relevant genes in the deletion region with functions, phenotypes and cases with agreeable phenotypes**

| ***Gene*** | ***Name*** | ***OMIM*** | ***Band*** | ***Function*** | ***Phenotype*** | ***Cases*** |
| --- | --- | --- | --- | --- | --- | --- |
| *PDGFC* | Platelet-derived growth factor C | 608452 | q32.1 | Growth factor for embryonic fusion of the palate shelf [23] | Cleft lip, cleft palate | [7], [10] |
| *TLL1* | Tolloid-like 1 | 606742 | q32.3 | Important for the development of the mammalian heart, specifically for interatrial septum [25] | Atrial septal defect | [6], [7], [9] #16 and #17, [10], [13], [15], [16], [17], [18], [19], [21], DECIPHER #276704 |
| *SCRG1* | Stimulator of chondrogenesis 1 | 603163 | q34.1 | Associated with neurodegenerative changes | Bipolar disorder and schizophrenia, of possible interest for intellectual disability | [6], [9] case #13, DECIPHER #264122, #276704, #249192, #254882, #267783, and #251175 |
| *HAND2* | Heart- and neural crest derivatives-expressed 2 | 602407 | q34.1 | Cardiac morphogenesis, angiogenesis, formation of right ventricle and aortic arch arteries; also implicated in limb development [27, 28] | Congenital heart defect, aortic arch artery deformities, limb developmental defects | [6], [7], [9] #16 and #17, [10], [13], [15], [16], [17], [18], [19], [21], DECIPHER #276704 |
| *CASP3* | Caspase 3 | 600636 | q35.1 | Important for maintaining spiral ganglion neurons and support of inner and outer hair cells [40] | Hearing loss | [16], DECIPHER #256186, present case |
| *PDLIM3* | PDZ and LIM domain protein 3 | 605889 | q35.1 | Cytoskeletal assembly via assembly of alpha-actin complexes, morphogenesis of the right ventricular chamber [29] | Right ventricular cardiomyopathy | [7], [9] #16, [17] |
| *SORBS2* | Sorbin and SH3 domain containing 2 | - | q35.1 | Subcellular localization in epithelial and cardiac tissue; though to act in cytoskeletal organization [30] | Congenital heart defect, cleft palate | [6], [9] #17 and #20, [13], [15], [16], [19], DECIPHER #276704, present case |
| *KLKB1* | Kallikrein B, plasma 1 | 229000 | q35.2 | Blood coagulation pathway, regulates blood pressure [37] | Fletcher factor deficiency | Present case |
| *F11* | Coagulation factor XI | 264900 | q35.2 | Blood coagulation pathway [36] | Factor XI deficiency | Present case |
| *FAT1* | FAT tumour suppressor 1 | 600976 | q35.2 | Developmental cell proliferation control, highly expressed during cardiac and vascular remodelling [34] | Implicated in bipolar affective [32] and autism spectrum disorder [33] | [20], [22], DECIPHER #249536 and #256186 |
