# Supplementary material for: Terminal chromosome 4q deletion syndrome in an infant with hearing impairment and moderate syndromic features: review of literature
Source: BMC Med Genet. 2014 Jun 25;15:72. doi: 10.1186/1471-2350-15-72 (PMC4077152; doi:10.1186/1471-2350-15-72)
Supplement: Additional file 2: Table S2 — Summary of our proband and cases from DECIPHER and the literature with deletions exclusively residing in the 4q31.1qter region. [file 1471-2350-15-72-S2.doc]

**Table S2 Summary of our proband and cases from DECIPHER and the literature with deletions exclusively residing in the 4q31.1qter region**

| ***Case*** | ***Sex*** | ***Size (Mb)*** | ***Origin*** | ***CHD*** | ***EA*** | ***HL*** | ***CP*** | ***CO*** | ***SD*** | ***GD*** | ***ID*** | ***ASD*** | ***BD*** | ***MH*** | ***CJF*** | ***HFA*** | ***UA*** | ***WID*** | ***UGA*** | ***PRS*** | ***SE*** | ***RI*** |
| --- | --- | --- | --- | --- | --- | --- | --- | --- | --- | --- | --- | --- | --- | --- | --- | --- | --- | --- | --- | --- | --- | --- |
| DECIPHER #253743 | f | 4.9 | de novo | + |  |  |  |  |  |  |  |  |  |  |  |  |  |  |  |  |  |  |
| DECIPHER #249541 | nd | 3.4 | nd |  | + |  |  |  |  |  | + |  |  |  | + |  |  |  |  |  |  |  |
| [7] | f | 40.1^*^ | de novo | + | + |  | + |  |  | + |  |  |  |  | + | + |  |  |  |  |  |  |
| DECIPHER #269176 | f | 6.9 | de novo |  |  |  |  |  |  |  | + |  |  | + | + |  |  |  |  |  |  | + |
| [10] | f | 27.6^*^ | de novo | + |  |  | + |  |  | + |  |  |  |  | + | + | + |  |  | + |  |  |
| DECIPHER #264122 | f | 32.6 | de novo |  |  |  |  |  |  |  | + |  |  |  |  |  |  |  |  |  |  |  |
| [18] | m | 21.0^*^ | de novo | + | + |  | + |  | + |  |  |  |  |  | + | + |  |  |  |  |  | + |
| [9] #13 | m | 24.9 | nd |  | + |  | + |  | + | + | + |  |  |  | + | + | + |  | + |  |  | + |
| [17] | f | 25.7 | de novo | + | + |  |  |  |  | + |  |  |  |  | + | + |  |  | + |  |  |  |
| DECIPHER #264942 | f | 10.0 | nd |  |  |  |  |  |  | + |  |  |  |  |  | + |  |  |  |  |  |  |
| DECIPHER #257358 | f | 5.4 | inherited |  |  |  |  |  |  |  | + |  |  |  | + |  |  |  |  |  |  |  |
| [9] #16 | f | 24.5 | de novo | + |  |  |  |  |  | + |  |  |  |  | + | + |  |  |  |  |  |  |
| [21] | m | 11.6 | de novo | + | + |  |  |  |  | + |  |  |  |  |  | + |  |  |  |  |  |  |
| [19] | m | 18.9-22.9^*^ | de novo | + | + |  | + |  |  | + |  |  | + | + | + | + |  |  | + |  |  |  |
| [14] | f | 21.1^*^ | de novo |  |  |  | + |  |  |  |  |  |  |  | + | + | + |  |  | + |  |  |
| [16] | m | 21.1^*^ | de novo | + | + | + | + | + |  | + |  |  |  | + | + | + |  | + |  |  |  |  |
| [13] | f | 21.1^*^ | de novo | + |  |  |  |  |  | + |  |  |  |  | + | + |  |  |  |  |  |  |
| [15] | m | 21.1^*^ | de novo | + | + |  |  |  |  |  |  |  | + |  | + | + |  |  |  |  |  |  |
| DECIPHER #249536 | nd | nd | nd |  | + |  | + |  |  |  |  | + |  |  | + |  |  | + | + |  |  |  |
| DECIPHER #276704 | f | 19.8 | de novo | + | + |  |  |  | + | + | + |  |  |  | + | + |  |  |  |  |  |  |
| DECIPHER #249192 | f | 18.0 | nd |  |  |  | + |  |  |  | + |  |  |  |  |  | + |  |  |  |  |  |
| DECIPHER #254882 | m | 1.8 | nd |  |  |  |  |  |  |  | + |  |  |  | + |  |  |  |  |  | + |  |
| DECIPHER #267783 | m | 1.1 | inherited |  |  |  |  |  |  |  | + |  | + |  |  |  |  |  |  |  | + |  |
| DECIPHER #251175 | f | 1.4 | de novo |  |  |  |  |  |  |  | + |  |  |  | + |  |  |  |  |  |  |  |
| [6] | f | 16.4 | de novo | + |  |  |  |  |  | + | + |  | + |  | + | + |  |  |  | + |  |  |
| [9] #17 | f | 14.6 | nd | + | + |  | + |  |  | + |  |  |  | + | + |  |  |  |  |  |  |  |
| DECIPHER #256186 | m | 10.0 | inherited |  |  | + |  | + | + |  | + | + |  |  | + |  |  |  |  |  |  |  |
| DECIPHER #249476 | nd | 8.5 | nd |  |  |  |  |  |  |  | + |  |  | + | + |  |  |  |  |  |  |  |
| DECIPHER #249458 | nd | 7.7 | nd |  |  |  |  |  |  |  |  |  |  | + | + |  |  |  |  |  |  |  |
| [20] | m | 6.8 | de novo |  |  |  |  |  | + |  |  | + |  | + |  |  |  |  |  |  |  |  |
| Present case | m | 6.9 | de novo | + |  | + | + | + | + |  |  |  |  |  |  |  |  |  |  |  |  |  |
| DECIPHER #248967 | f | 1.0 | inherited |  |  |  | + |  |  |  |  |  |  |  |  |  |  |  |  |  |  |  |
| [9], #20, F | f | 0.5 | nd | + | + |  | + |  |  |  |  |  |  | + | + | + |  |  |  |  |  | + |
| DECIPHER #249655 | nd | 2.6 | nd |  |  |  |  |  |  |  | + |  | + |  | + |  |  |  |  |  |  |  |
| [22] | m | 2.1 | nd | + |  |  |  |  | + | + | + | + | + |  |  |  |  |  |  |  |  |  |
| DECIPHER #278055 | f | 0.9 | inherited |  |  |  |  |  |  |  |  |  | + |  |  |  |  |  |  |  |  |  |

Abbreviations: nd = not disclosed; f = female; m = male; CHD = congenital heart defect; EA = ear abnormalities; HL = hearing loss; CP = cleft palate; CO = cryptorchidism; SD = speech delay; GD = general delay; BD = behavioural disorder; MH = muscle hypotonia; CJF = craniofacial, jaw, facial dysmorphism; HFA = hand and/or foot abnormalities; UA = ulna absent; WID = wide intermamillary distance; UGA = urogenital abnormalities; PRS = Pierre Robin sequence; SE = seizures or epilepsy; RI = recurrent infection.

“+” sign denotes the phenotype is present.

*estimated size based on chromosomal band assignment from FISH, GTG-Banding or BAC aCGH.
